# Improved Guided Image Fusion for Magnetic Resonance and Computed Tomography Imaging

**DOI:** 10.1155/2014/695752

**Published:** 2014-02-13

**Authors:** Amina Jameel, Abdul Ghafoor, Muhammad Mohsin Riaz

**Affiliations:** ^1^Department of Electrical Engineering, College of Signals, National University of Sciences and Technology (NUST), Islamabad 46000, Pakistan; ^2^Center for Advanced Studies in Telecommunication (CAST), COMSATS Institute of Information Technology, Islamabad, Pakistan

## Abstract

Improved guided image fusion for magnetic resonance and computed tomography imaging is proposed. Existing guided filtering scheme uses Gaussian filter and two-level weight maps due to which the scheme has limited performance for images having noise. Different modifications in filter (based on linear minimum mean square error estimator) and weight maps (with different levels) are proposed to overcome these limitations. Simulation results based on visual and quantitative analysis show the significance of proposed scheme.

## 1. Introduction

Medical images from different modalities reflect different levels of information (tissues, bones, etc.). A single modality cannot provide comprehensive and accurate information [1, 2]. For instance, structural images obtained from magnetic resonance (MR) imaging, computed tomography (CT), and ultrasonography, and so forth, provide high-resolution and anatomical information [1, 3]. On the other hand, functional images obtained from position emission tomography (PET), single-photon emission computed tomography (SPECT), and functional MR imaging, and so forth, provide low-spatial resolution and functional information [3, 4]. More precisely, CT imaging provides better information on denser tissue with less distortion. MR images have more distortion but can provide information on soft tissue [5, 6]. For blood flow and flood activity analysis, PET is used which provide low space resolution. Therefore, combining anatomical and functional medical images through image fusion to extract much more useful information is desirable [5, 6]. Fusion of CT/MR images combines anatomical and physiological characteristics of human body. Similarly fusion of PET/CT is helpful for tumor activity analysis [7].

Image fusion is performed on pixels, features, and decision levels [8–10]. Pixel-level methods fuse at each pixel and hence reserve most of the information [11]. Feature-level methods extract features from source images (such as edges or regions) and combine them into a single concatenated feature vector [12, 13]. Decision-level fusion [11, 14] comprises sensor information fusion, after the image has been processed by each sensor and some useful information has been extracted out of it.

Pixel-level methods include addition, subtraction, division, multiplication, minimum, maximum, median, and rank as well as more complicated operators like Markov random field and expectation-maximization algorithm [15]. Besides these, pixel level also includes statistical methods (principal component analysis (PCA), linear discriminant analysis, independent component analysis, canonical correlation analysis, and nonnegative matrix factorization). Multiscale transforms like pyramids and wavelets are also types of pixel-level fusion [11, 14]. Feature-level methods include feature based PCA [12, 13], segment fusion [13], edge fusion [13], and contour fusion [16]. They are usually robust to noise and misregistration. Weighted decision methods (voting techniques) [17], classical inference [17], Bayesian inference [17], and Dempster-Shafer method [17] are examples of decision-level fusion methods. These methods are application dependent; hence, they cannot be used generally [18].

Multiscale decomposition based medical image fusion decompose the input images into different levels. These include pyramid decomposition (Laplacian [19], morphological [20], and gradient [21]); discrete wavelet transform [22]; stationary wavelet transform [23]; redundant wavelet transform [24]; and dual-tree complex wavelet transform [25]. These schemes produce blocking effects because the decomposition process is not accompanied by any spatial orientation selectivity.

To overcome the limitations, multiscale geometric analysis methods were introduced for medical image fusion. Curvelet transform based fusion of CT and MR images [26] does not provide a proper multiresolution representation of the geometry (as curvelet transform is not built directly in the discrete domain) [27]. Contourlet transform based fusion improves the contrast, but shift-invariance is lost due to subsampling [27, 28]. Nonsubsampled contourlet transform with a variable weight for fusion of MR and SPECT images has large computational time and complexity [27, 29].

Recently, guided filter fusion (GFF) [30] is used to preserve edges and avoid blurring effects in the fused image. Guided filter is an edge-preserving filter and its computational time is also independent of filter size. However, the method provides limited performance for noisy images due to the use of Gaussian filter and two-level weight maps. An improved guided image fusion for MR and CT imaging is proposed to overcome these limitations. Simulation results based on visual and quantitative analysis show the significance of proposed scheme.

## 2. Preliminaries

In this section, we briefly discuss the methodology of GFF [30]. The main steps of the GFF method are filtering (to obtain the two-scale representation), weight maps construction, and fusion of base and detail layers (using guided filtering and weighted average method).

Let *F* be the fused image obtained by combining input images *A* and *B* of same sizes (*M* × *N*). The base (*I*_11_ and *I*_12_) and detail (*I*_21_ and *I*_22_) layers of source images are

(1) $$\begin{array}{c} \left[\begin{array}{cc} I_{11} & I_{12}\\ I_{21} & I_{22} \end{array}\right]=\left[\begin{array}{cc} A*f & B*f\\ A-A*f & B-B*f \end{array}\right]\text{,} \end{array}$$

where *f* is the average filter. The base and detail layers contain large- and small-scale variations, respectively. The saliency images are obtained by convolving *A* and *B* and with a Laplacian filter *h* followed by a Gaussian filter *g*; that is,

(2) $$\begin{array}{c} \left[\frac{S_{1}}{S_{2}}\right]=\left[\frac{\left|A*h\right|*g}{\left|B*h\right|*g}\right]. \end{array}$$

The weight maps *P*_1_ and *P*_2_ are

(3) $$\begin{array}{c} \left[\frac{P_{1}}{P_{2}}\right]=\left[\frac{\xi \left(S_{1},S_{2}\right)}{\xi \left(S_{2},S_{1}\right)}\right], \end{array}$$

where *ξ*(*S*_1_, *S*_2_) is a function with value 1 for *S*_1_(*m*, *n*) ≥ *S*_2_(*m*, *n*) and value 0 for *S*_1_(*m*, *n*) < *S*_2_(*m*, *n*) (similarly for *ξ*(*S*_2_, *S*_1_)). *S*_1_(*m*, *n*) and *S*_2_(*m*, *n*) are the saliency values for (*m*, *n*) pixel in *A* and *B*, respectively.

Guided image filtering is performed to obtain the refined weights *W*_11_, *W*_12_, *W*_21_, and *W*_22_ as

(4) $$\begin{array}{c} \left[\begin{array}{cc} W_{11} & W_{12}\\ W_{21} & W_{22} \end{array}\right]=\left[\begin{array}{cc} G_{r_{1},\epsilon_{1}}\left(P_{1},A\right) & G_{r_{2},\epsilon_{2}}\left(P_{1},A\right)\\ G_{r_{1},\epsilon_{1}}\left(P_{2},B\right) & G_{r_{2},\epsilon_{2}}\left(P_{2},B\right) \end{array}\right], \end{array}$$

where *r*_1_, *ϵ*_1_, *r*_2_, and *ϵ*_2_ are the parameters of the guided filter *W*_11_ and *W*_21_ and *W*_12_ and *W*_22_ are the base layer and the detail layer weight maps.

The fused image *F* is obtained by weighted averaging of the corresponding layers; that is,

(5) $$\begin{array}{c} F=\overset{2}{\underset{i_{1}=1}{\sum }}\;‍\overset{2}{\underset{i_{2}=1}{\sum }}W_{i_{1}i_{2}}I_{i_{2}i_{1}}. \end{array}$$

The major limitations of GFF [30] scheme are summarized as follows.

1. The Gaussian filter from (2) is not suitable for Rician noise removal. Thus, the algorithm has limited performance for noisy images. Hence filter of (2) needs to be modified to incorporate noise effects.
2. The weight maps *P*_1_ and *P*_2_ from (3) can be improved by defining more levels. The main issue with binary assignment (0 and 1) is that when the saliency values are approximately equal, the effect of one value is totally discarded, which results in degraded fused image.

## 3. Proposed Methodology

The proposed scheme follows the methodology of GFF [30] with necessary modifications to incorporate the above listed limitations. This section first discusses the modification proposed due to noise artifacts and then the improved weight maps are presented.

### 3.1. Improved Saliency Maps

The acquired medical images are usually of low quality (due to artifacts), which degrade the performance (both in terms of human visualization and quantitative analysis).

Beside other artifacts, MR images often contain Rician Noise (RN) which causes random fluctuations in the data and reduces image contrast [31]. RN is generated when real and imaginary parts of MR data are corrupted with zero-mean, equal variance uncorrelated Gaussian noise [32]. RN is a nonzero mean noise. Note that the noise distribution tends to Rayleigh distribution in low intensity regions and to a Gaussian distribution in regions of high intensity of the magnitude image [31, 32].

Let $\dot{A}=A+N_{R}$ be image obtained using MR imaging containing Rician noise *N*_*R*_. The CT image *B* has higher spatial resolution and negligible noise level [33, 34].

The source images are first decomposed into base ${\dot{I}}_{11}$ and *I*_12_ and detail ${\dot{I}}_{21}$ and *I*_22_ layers following (1):

(6) $$\begin{array}{c} \left[\begin{array}{cc} {\dot{I}}_{11} & I_{12}\\ {\dot{I}}_{21} & I_{21} \end{array}\right]=\left[\begin{array}{cc} \dot{A}*f & B*f\\ \dot{A}-\dot{A}*f & B-B*f \end{array}\right]. \end{array}$$

${\dot{I}}_{11}$ and ${\dot{I}}_{21}$ have an added noise term compared to (1). Linear minimum mean square error estimator (LMMSE) is used instead of Gaussian filter for minimizing RN, consequently improving fused image quality.

The saliency maps ${\dot{S}}_{1}$ and ${\dot{S}}_{2}$ are thus computed by applying the LMMSE based filter *q* and following (2):

(7) $$\begin{array}{c} \left[\frac{{\dot{S}}_{1}}{{\dot{S}}_{2}}\right]=\left[\frac{\left|A*h\right|*q+\left|N_{R}*h\right|*q}{\left|B*h\right|*q}\right]. \end{array}$$

The main purpose of *q* is to make the extra term *N*_*R*_∗*h* as in ${\dot{S}}_{1}$ small as possible while enhancing the image details.

### 3.2. Improved Weight Maps

The saliency maps are linked with detail information in the image. The main issue with 0 and 1 weight assignments arises in GFF [30] when different images have approximately equal saliency values. In such cases, one value is totally discarded. For noisy MR images, the saliency value may be higher at a pixel due to noise; in that case it will assign value 1 (which is not desirable). An appropriate solution is to define a range of values for weight maps construction.

Let Δ_*AB*_ = *S*_1_(*m*, *n*) − *S*_2_(*m*, *n*), and *S*_1_(*m*, *n*) ≥ *S*_2_(*m*, *n*),

(8) $$\begin{array}{c} {\dot{P}}_{1}\left(m,n\right)=\{\begin{array}{cc} 1 & \text{if}\;\Delta_{AB}\geq 0.3\\ 0.8 & \text{if}\;\Delta_{AB}\geq 0.25\\ 0.7 & \text{if}{\;\Delta }_{AB}\geq 0.2\\ 0.6 & \text{if}\;\Delta_{AB}\geq 0.15\\ 0 & \text{otherwise}. \end{array} \end{array}$$

Let Δ_*BA*_ = *S*_2_(*m*, *n*) − *S*_1_(*m*, *n*), and *S*_2_(*m*, *n*) ≥ *S*_1_(*m*, *n*),

(9) $$\begin{array}{c} {\dot{P}}_{2}\left(m,n\right)=\{\begin{array}{cc} 1 & \text{if}\;\Delta_{BA}\geq 0.3\\ 0.8 & \text{if}{\;\Delta }_{BA}\geq 0.25\\ 0.7 & \text{if}\;\Delta_{BA}\geq 0.2\\ 0.6 & \text{if}{\;\Delta }_{BA}\geq 0.15\\ 0 & \text{otherwise}. \end{array} \end{array}$$

**Figure 1:**
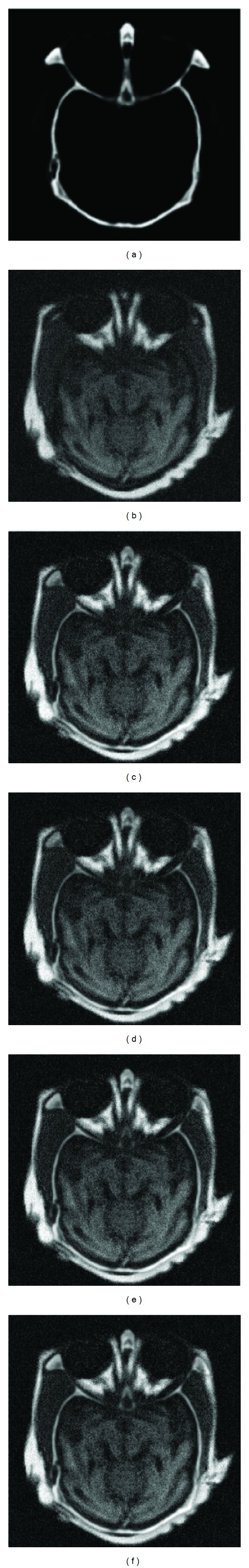
Weight maps comparison: (a) CT image, (b) noisy MR image, (c) fused image with 3 weight maps, (d) fused image with 4 weight maps, (e) fused image with 5 weight maps, and (f) fused image with 6 weight maps.

These values are selected empirically and may be further adjusted to improve results. Figures 1(a) and 1(b) show CT and noisy MR images, respectively. Figures 1(c)–1(f) show the results of applying different weights. The information in the upper portion of the fused image increases as more levels are added to the weight maps.

The weight maps are passed through guided filter to obtain ${\dot{W}}_{11}$, ${\dot{W}}_{21}$, ${\dot{W}}_{12}$, and ${\dot{W}}_{22}$. Finally the fused image $\dot{F}$ is

(10) $$\begin{array}{c} \dot{F}=\overset{2}{\underset{i_{1}=1}{\sum }}\;\overset{2}{\underset{i_{2}=1}{\sum }}{\dot{W}}_{i_{1}i_{2}}{\dot{I}}_{i_{2}i_{1}}. \end{array}$$

LMMSE based filter reduces the Rician noise and the more levels of weight maps ensure that more information is transferred to the fused image. The incorporation of the LMMSE based filter and a range of weight map values makes the proposed method suitable for noisy images.

## 4. Results and Analysis

The proposed method is tested on several pairs of source (MR and CT) images. For quantitative evaluation, different measures including mutual information (MI) [35] measure *ζ*_MI_, structural similarity (SSIM) [36] measure *ζ*_SSIM_, Xydeas and Petrović's [37] measure *ζ*_XP_, Zhao et al.'s [38] measure *ζ*_Z_, Piella and Heijmans's [39] measures *ζ*_PH_1__ and *ζ*_PH_2__, and visual information fidelity fusion (VIFF) [40] metric *ζ*_VIFF_ are considered.

### 4.1. MI Measure

MI is a statistical measure which provides the degree of dependencies in different images. Large value of MI implies better quality and vice versa [11, 33, 35]:

(11) ζMI=2[1(HA+HF)∑a,fPAF(a,f)log⁡PAF(a,f)PA(a)PF(f)   +1(HB+HF)∑b,fPBF(b,f)log⁡PBF(b,f)PB(b)PF(f)],

where *H*_*A*_, *H*_*B*_, and *H*_*F*_ are the entropies of *A*, *B*, and *F* images, respectively. *P*_*AF*_ is the jointly normalized histogram of *A* and *F*, *P*_*BF*_ is the jointly normalized histogram of *B* and *F*, and *P*_*A*_, *P*_*B*_, and *P*_*F*_ are the normalized histograms of *A*, *B*, and *F*, respectively.

### 4.2. SSIM [36] Measure

SSIM [36] measure is defined as

(12) ζSSIM(A,B,F) ={λwζSSIM(Aw,Fw)+(1−λw)ζSSIM(Bw,Fw) if  ζSSIM(Aw,Bw ∣ w)≥0.75max⁡(ζSSIM(Aw,Fw),ζSSIM(Bw,Fw)) if  ζSSIM(Aw,Bw ∣ w)<0.75,

where *w* is a sliding window and *λ*(*w*) is

(13) $$\begin{array}{c} \lambda_{w}=\frac{\sigma_{A_{w}}}{\sigma_{A_{w}}+\sigma_{B_{w}}}, \end{array}$$

where *σ*_*A*_*w*__ and *σ*_*B*_*w*__ are the variance of images *A* and *B*, respectively.

### 4.3. Xydeas and Petrović's [37] Measure

Xydeas and Petrović [37] proposed a metric to evaluate the amount of edge information, transferred from input images to fused image. It is calculated as

(14) ζXP=∑m=1N∑n=1M(QAF(m,n)τA(m,n)+QBF(m,n)τB(m,n)) ×(∑m=1N∑n=1M(τA(m,n)+τB(m,n)))−1,

where *Q* ^*AF*^ and *Q* ^*BF*^ are the product of edge strength and orientation preservation values at location (*m*, *n*), respectively. The weights *τ* ^*A*^(*m*, *n*) and *τ* ^*B*^(*m*, *n*) reflect the importance of *Q* ^*AF*^(*m*, *n*) and *Q* ^*BF*^(*m*, *n*), respectively.

### 4.4. Zhao et al.'s [38] Metric

Zhao et al. [38] used the phase congruency (provides an absolute measure of image feature) to define an evaluation metric. The larger value of the metric describes a better fusion result. The metric *ζ*_Z_ is defined as the geometric product of phase congruency, maximum and minimum moments, respectively.

### 4.5. Piella and Heijmans's [39] Metric

Piella and Heijmans's [39] metrics *ζ*_*P*_1__ and *ζ*_*P*_2__ are defined as

(15) ζP1=1|W|∑w∈W[λ(w)Qo(A,F ∣ w)+(1−λ(w))Qo(B,F ∣ w)]ζP2=∑w∈Wc(w)[λ(w)Qo(A,F ∣ w)+(1−λ(w))Qo(B,F ∣ w)],

where *Q*_*o*_(*A*, *F* | *w*) and *Q*_*o*_(*B*, *F* | *w*) are the local quality indexes calculated in a sliding window *w* and *λ*(*w*) is defined as in (13). Consider

(16) $$\begin{array}{c} Q_{o}\left(A,F\;|\;w\right)=\frac{4\sigma_{AF}\overset{-}{A}\;\overset{-}{F}}{\left({\overset{-}{A}}^{2}+{\overset{-}{F}}^{2}\right)\left(\sigma_{A}^{2}+\sigma_{F}^{2}\right)}, \end{array}$$

where $\overset{-}{A}$ is the mean of *A* and *σ*_*A*_^2^ and *σ*_*AF*_ are the variance of *A* and the covariance of *A*, *B*, respectively. Consider

(17) $$\begin{array}{c} c_{w}=\frac{\max\left[\sigma_{A_{w}},\sigma_{B_{w}}\right]}{\sum_{w^{\prime }\in W}^{}\left[\sigma_{A_{w}^{\prime }},\sigma_{A_{w}^{\prime }}\right]}, \end{array}$$

where *σ*_*A*_*w*__ and *σ*_*B*_*w*__ are the variance of images *A* and *B* within the window *w*, respectively.

### 4.6. VIFF [40] Metric

VIFF [40] is a multiresolution image fusion metric used to assess fusion performance objectively. It has four stages. (1) Source and fused images are filtered and divided into blocks. (2) Visual information is evaluated with and without distortion information in each block. (3) The VIFF of each subband is calculated and the overall quality measure is determined by weighing (of VIFF at different subbands).

**Figure 2:**
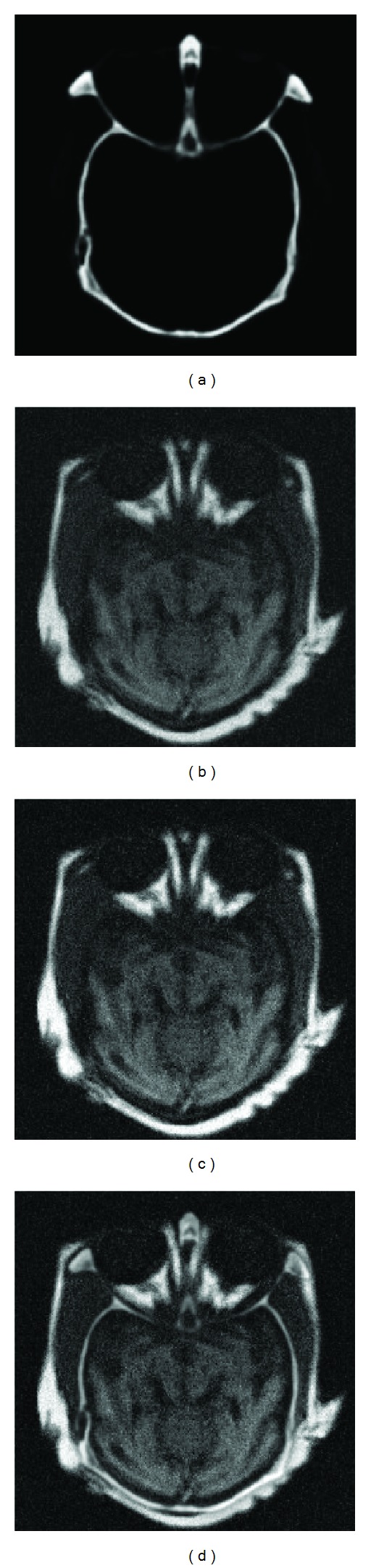
(a) CT image, (b) noisy MR image, (c) GFF [30] fused image, and (d) proposed fused image.

Figure 2 shows a pair of CT and MR images. It can be seen that the CT image (Figure 2(a)) provides clear bones information but no soft tissues information, while in contrast to CT image the MR image in Figure 2(b) provides soft tissues information. The fused image must contain both the information of bones and soft tissues. The fused image obtained using proposed scheme in Figure 2(d) shows better results as compared to fused image obtained by GFF [30] in Figure 2(c).

**Figure 3:**
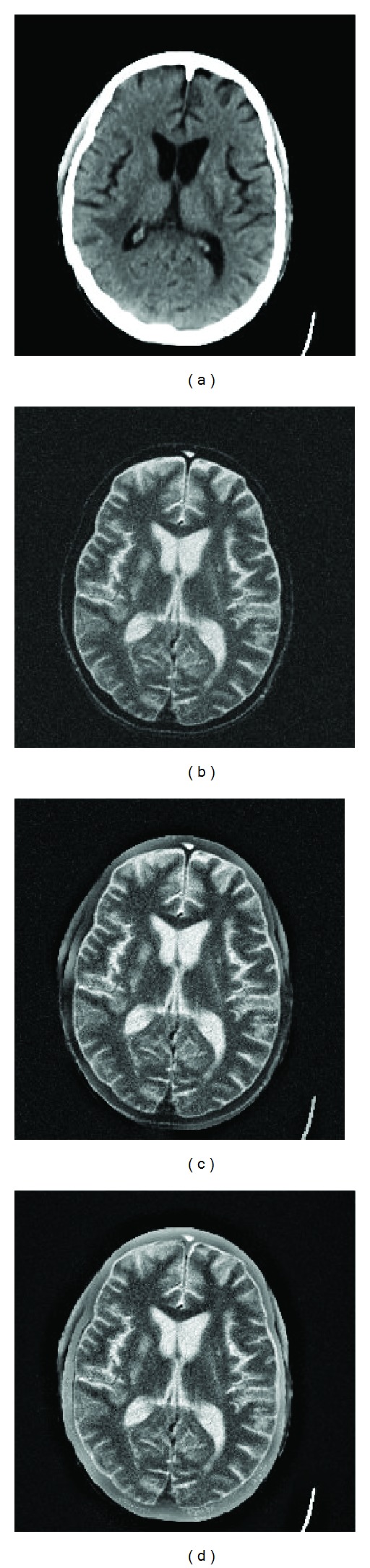
(a) CT image, (b) noisy MR image, (c) GFF [30] fused image, and (d) proposed fused image.

Figure 3 shows the images of a patient suffering from cerebral toxoplasmosis [41]. A more comprehensive information consisting of both the CT and MR images is the requirement in clinical diagnosis. The improvement in fused image using proposed scheme can be observed in Figure 3(d) compared to image obtained by GFF [30] in Figure 3(c).

**Figure 4:**
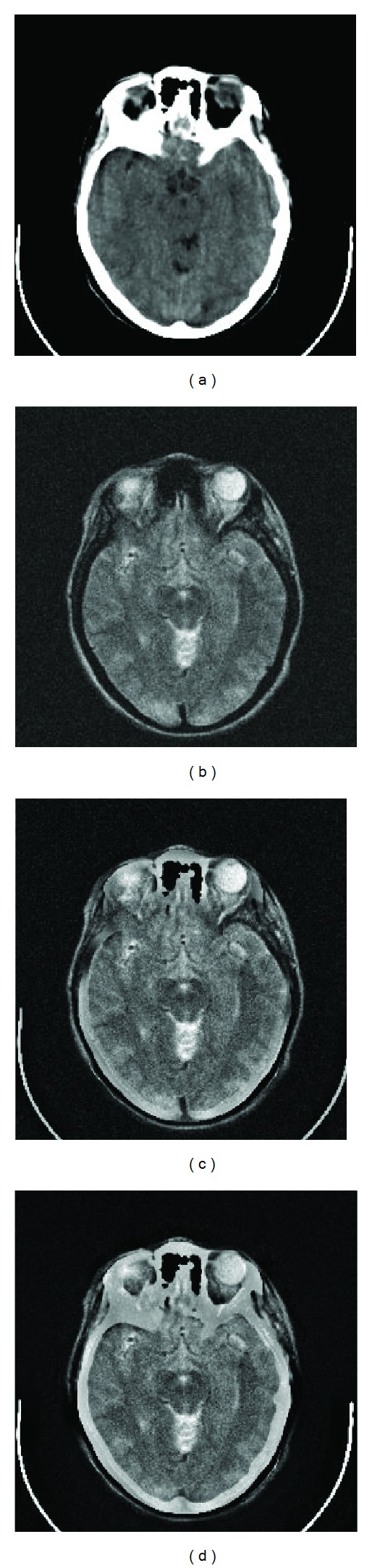
(a) CT image, (b) noisy MR image, (c) GFF [30] fused image, and (d) proposed fused image.

Figure 4 shows a pair of CT and MR images of a woman suffering from hypertensive encephalopathy [41]. The improvement in fused image using proposed scheme can be observed in Figure 4(d) compared to image obtained by GFF [30] in Figure 4(c).

**Figure 5:**
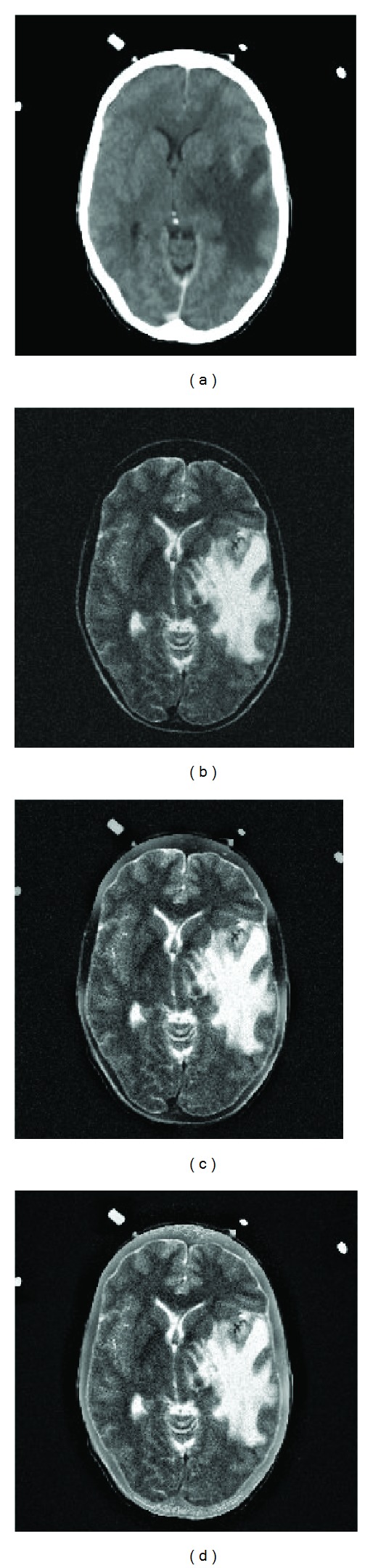
(a) CT image, (b) noisy MR image, (c) GFF [30] fused image, and (d) proposed fused image.

Figure 5 shows a pair of images containing acute stroke disease [41]. The improvement in quality of fused image obtained using proposed scheme can be observed in Figure 5(d) compared to Figure 5(c) (image obtained by GFF [30]).

Table 1 shows that proposed scheme provides better quantitative results in terms of *ζ*_MI_, *ζ*_SSIM_, *ζ*_XP_, *ζ*_Z_, *ζ*_*P*_1__, *ζ*_*P*_2__, and *ζ*_VIFF_ as compared to GFF [30] scheme.

## 5. Conclusions

An improved guided image fusion for MR and CT imaging is proposed. Different modifications in filter (LMMSE based) and weights maps (with different levels) are proposed to overcome the limitations of GFF scheme. Simulation results based on visual and quantitative analysis show the significance of proposed scheme.

## Figures and Tables

**Table 1:** Quantitative analysis of GFF [30] and proposed schemes.

Quantitative measures	Example 1		Example 2		Example 3		Example 4	
	GFF [30]	Proposed	GFF [30]	Proposed	GFF [30]	Proposed	GFF [30]	Proposed
*ζ*_MI_	0.2958	0.2965	0.4803	0.5198	0.4164	0.4759	0.4994	0.5526
*ζ*_SSIM_	0.3288	0.3540	0.3474	0.3519	0.3130	0.3139	0.2920	0.2940
*ζ*_XP_	0.4034	0.5055	0.4638	0.4678	0.4473	0.4901	0.4498	0.4653
*ζ*_Z_	0.1600	0.1617	0.3489	0.3091	0.2061	0.2193	0.3002	0.2855
*ζ*_*P*_1__	0.4139	0.4864	0.2730	0.3431	0.2643	0.3247	0.2729	0.3339
*ζ*_*P*_2__	0.4539	0.7469	0.5188	0.6387	0.6098	0.7453	0.5268	0.6717
*ζ*_VIFF_	0.2561	0.3985	0.1553	0.2968	0.1852	0.3009	0.1842	0.3487
